# MED2/342: The Role of the Internet in the Education of Health Information Managers in Courses in the USA, Australia, Germany and China

**DOI:** 10.2196/jmir.1.suppl1.e45

**Published:** 1999-09-19

**Authors:** A Lange

**Affiliations:** ^1^The University of SydneyLidcombeAustralia

**Keywords:** Education, Health Information Management, Computer Literacy, Email, Allied Health Professionals

## Abstract

**Introduction, Methods:**

Health Information Managers (HIMs) are allied health professionals educated to manage health information. They work in medical record departments, coding institutions, hospital administration, health planning institutions, hospital information departments, clinical trials, pharmaceutical companies and IT companies for the health sector. Education of HIMs at universities and other tertiary institutions contains around 25% of all subjects that are oriented towards 'core' information technology, with another 50% of all subjects enhanced by the use of the Internet. This paper discusses the impact of Internet use by HIM students in four countries: the USA with around 650 Registered Record Administrators and 1600 Accredited Record Technicians graduating per year; Australia with around 80 HIMs graduating per year; Germany with around 500 Medical Documentalists graduating per year; China with an estimated 400 Medical Record Administrators graduating per year. Overall the IT equipment, in the form of computer labs for HIM education, is better in Germany and Australia than it is in the USA and China. In the USA, Australia and Germany every student has access to the Internet, using it for information exchange and literature-based searches. Germany has a slightly different teaching approach for Medical Documentalists, which concentrates more on pharmacology and literature searches about drugs. This is due to the fact that these graduates are primarily employed by pharmaceutical companies and very rarely by hospitals. German Schools provided traditionally access to literature databases, but use more and more the Internet for these searches. Schools in Australia are providing an email account for every student, while educators use and develop Internet-based teaching packages, which support face-to-face teaching.

**Examples:**

Packages to learn medical terminology, a 'health care game' to find out about the Australian Health care system at the School of HIM in Sydney, and demo-databases downloaded from the Inpatient Statistics Collection of New South Wales/Australia.

**Discussion:**

It is estimated that the students used the Internet to obtain about half of all references given when submitting essays or presentations relating to HIM topics in 1999, compared with around 5 % three years ago. Schools in the USA provide Internet access mainly through computer labs dedicated to general use for all students of the campus. China is just starting to use computers in education and no School is currently equipped with Internet access for all students. Major drawbacks of the intense Internet use are: a) the connection fees for Universities, and b) the difficulty to prevent students from downloading software that is "upsetting" the operating system of the computers they use.

**Conclusion:**

Overall the use of the Internet for HIM students has increased dramatically in the last 2 years, increasing the expenses for Internet access for the Universities in a time, when education is not longer on the priority list for Government budgets.

